# What Says the Student?

**Published:** 1890-11-01

**Authors:** 


The Hospital

A Weekly Journal of

Science, Medicine, Nursing, and Philanthropy.

*For Hospitals, Asylums, Infirmaries, Dispensaries, Medical Practitioners, Students, Nurses, and the Charitable Public.*

Vol. IX.—No. 214.] [November 1, 1890.

### What Says the Student?

This is the day of examiners in medicine and other sciences; the day of teachers has yet to dawn. Examiners are probably necessary at certain stages of progress, as the clearer of the forest must always precede the ploughman, the sower, and the reaper. But the work of the examiner constantly decreases in importance, and that of the teacher as steadily increases, as culture and character advance. The too great exaltation of the examiner tends to the depression of the teacher and the elevation of the crammer: the result being the dwarfing of the general mind and the extinction of the deeper culture. The immediate prospect of the young man who has chosen medicine for his life work is not too happy. He is brought into rich pastures of varied scientific study, and if he could eat with wise and ordered discretion he would in due time develop into a free-moving, active, and healthy master of scientific knowledge and aptitude; but there are examiners sitting at the other side of those fields of science, and many of them judge medical students as agricultural societies judge bullocks:—not by their power of active and graceful movement, but by their weight avoirdupois. So soon, therefore, as the student enters the field of study he is set upon by the teacher, who, at the bidding of the examiner, abdicates his teaching functions and assumes those of a crammer. From the first day of the medical student's career to the last, most of his teachers stuff him month by month, with the one sole object of making him fat enough to be placed at the examiners' table.

Teaching is a very young art among the professors of medical learning. That the majority of practising medical men have never been taught is proved in a very unhappy way from time to time by the sorry and discreditable figures which they cut in courts of law. What do we see there? A lawyer who knows absolutely nothing of medicine, or of the sciences on which it is based, will spend a couple of hours in reading up a particular medical case, and then with the utmost ease will make a medical man, even though he call himself a consultant, appear ridiculous to the whole world in his own special subject. If medical men had been accustomed from their earliest professional life to reason about the facts of science as well as to carry them in the memory, such things would not be possible. But reasoning freely and easily about the facts connected with the science and art of medicine seems to be the very last thing which the average medical man has the smallest capacity for.

What is it exactly that we mean by teaching? Does not the Medical Professor lecture and demonstrate, and is not that teaching? It may be; but it very seldom is. The average medical lecturer sets forth a series of facts in a more or less logical order, and when he has done that he thinks his work accomplished. But let him look at the question from the student's standpoint. When the latter has got the facts into his memory, is he educated—is he taught? As well may we say that we have turned a man into an artist because we have supplied him with brushes and paints, a square yard of canvas, and an easel. The whole subject can be seen at a glance. A medical student is given five years in which to prepare for his examinations. He is presented during that time with such enormous masses of facts which are entirely new to him that the only possible thing he can do with them is to put them, without criticism, into his memory, and to hope that they will stay there until he passes the final examination for his degree. Hundreds of young men during the present month and the month of November will commence for the first time this process of the wholesale gorging of unweighed, unmeasured, and partially understood facts. Is there any lecturer in any medical school in the kingdom who, after rational consideration, will call this process education?

But what is to be done to mend matters? The first thing is to see the present state of affairs in its true light, and to admit that it is bad. If we set before a man as much food for a single meal as would be abundantly sufficient for three, and compel him to eat it, can we be surprised if he falls ill? And do we need to ask twice what is the first step to be taken for his recovery? We must cut down the quantity of his food! All other remedies, however excellent will fail if that is neglected. In the case of the medical student, the first thing to be done is to diminish the extent of the ground he is called upon to cover, and the facts he is compelled to commit to memory. Can this be done without injury to the efficiency of the future medical man? It can be done with the greatest possible advantage to his efficiency. Here is a raw medical student of eighteen in the presence of the Professor of Chemistry. That professor will lecture to that student for the next six months exactly as if the boy's sole business were to become a specialist in chemistry. Nothing can be more absurd in itself, or unkind to the youth. What does he really want to know of chemistry? He wants to know generally the substances with which the science deals, and to understand clearly and intelligently the nature of chemical action; he needs to know more especially the facts about air, water, food, drugs, and physiological operations. If the scope of the chemist's lectures were limited to these subjects, and if the lecturer himself were a teacher and not a mere talker, and if he had for every dozen of his pupils a capable assistant teacher, then, with six months of instruction in the principles of chemistry, and three months of intelligent experimentation in the laboratory, every industrious medical student would become easily familiar with chemical action, and with the broad principles on which the great and wonderful science of chemistry is based. Thus trained thoroughly in the elements, a man could go forward at any period of his life into new fields of chemical knowledge with ever-increasing wonder and delight. But what is the case of the average medical man of the present time with regard to the science of chemistry? It is this—that he knows nothing whatever about it, and does not care to learn.

What has been said of chemistry may be affirmed with even more energy and propriety of botany and zoology. Is it desirable or possible that the average medical student should become a specialist in botany? And yet the average Professor of Botany lays himself out in his lectures to cover the whole field of botanical knowledge in six months; and, what is more, he insists upon his professional examinations covering the same or nearly the same ground. Now we admit to the full the value of elementary botanical knowledge to the medical man. But to attempt to convert a youth of eighteen or twenty into a botanist in six or nine months, whilst he is at the same time being turned into a chemist and a zoologist, is almost as ridiculous and futile as it would be to try to turn him into a chimpanzee or an elephant. This statement is proved in the most convincing way by the indisputable fact that the average medical man who has been two years in practice knows nothing whatever in an intelligent way either about chemistry, botany, or zoology.

We have left ourselves little space for offering a better scheme of education than that which is now established in this country. But we may say in a word that the reform which is demanded is that the sciences ancillary to medicine shall be taught in their elements at medical schools, not lectured about in their whole extent; and that the elements shall be taught and mastered thoroughly, as common arithmetic is taught and mastered before the higher mathematics are entered upon. Above all, the elements alone of those sciences must be given for examination. If this were done two results would follow: the student would be left with both time and mental energy for something like a thorough study of the structure and functions of the human body; of the diseases and accidents to which it is liable; and of the various methods of repair and cure which a rational experience has established. At present the human body and its diseases, as well as the curative arts, are studied in precisely the same smattering and inefficient way as the ancillary sciences. If the elements alone of botany, zoology, and chemistry were studied and examined in, whilst the sciences of man and of medicine were thoroughly and comprehensively taught, the average medical man, although he might not be a botanist, a zoologist, or a chemist, would at least be a physician. But the result of medical study as at present conducted is that the average young practitioner as he is turned out of the schools is neither a botanist, nor a zoologist, nor a chemist —nor a physician.

